# Effects of thyroxine and donepezil on hippocampal acetylcholine content, acetylcholinesterase activity, synaptotagmin-1 and SNAP-25 expression in hypothyroid adult rats

**DOI:** 10.3892/mmr.2014.2825

**Published:** 2014-10-30

**Authors:** FEN WANG, XIANZHONG ZENG, YANGBO ZHU, DAN NING, JUNXIA LIU, CHUNLEI LIU, XUEMEI JIA, DEFA ZHU

**Affiliations:** 1 Department of Endocrinology, Anhui Geriatric Institute, The First Affiliated Hospital of Anhui Medical University, Hefei, Anhui 230022, P.R. China; 2 Comprehensive Laboratory, College of Basic Medicine, Anhui Medical University, Hefei, Anhui 230032, P.R. China

**Keywords:** hypothyroidism, hippocampus, thyroxine, donepezil, acetylcholine, acetylcholinesterase, synaptotagmin-1, SNAP-25

## Abstract

A growing number of studies have revealed that neurocognitive impairment, induced by adult-onset hypothyroidism, may not be fully restored by traditional hormone substitution therapies, including thyroxine (T4). The present study has investigated the effect of T4 and donepezil (DON; an acetylcholinesterase (AChE) inhibitor) treatment on the hypothyroidism-induced alterations of acetylcholine (ACh) content and AChE activity. Furthermore, we examined synaptotagmin-1 (syt-1) and SNAP-25 expression in the hippocampus of adult rats. Adding 0.05% propylthiouracil to their drinking water for five weeks induced hypothyroidism in the rat models. From the fourth week, the rats were treated with T4, DON or a combination of both. Concentration of ACh and the activity of AChE was determined colorimetrically. The results demonstrated that hypothyroidism induced a significant decrease of Ach content and AChE activity (by 17 and 34%, respectively), which were restored to control values by T4 administration. DON treatment also restored Ach to the normal level. Protein levels of syt-1 and SNAP-25 were determined by immunohistochemistry. The results demonstrated that syt-1 was expressed at significantly lower levels in hypothyroid rats, while SNAP-25 levels were notably higher compared with the controls. Two-week treatment with T4 alone failed to normalize the expression levels of these two proteins, while co-administration of T4 and DON was able to induce this effect. These data suggested that the thyroid hormone, T4, may have a direct effect on the metabolism of hippocampal ACh in adult rats, and that the DON treatment may facilitate the recovery of synaptic protein impairments induced by hypothyroidism.

## Introduction

Adult-onset hypothyroidism causes a wide range of central nervous system dysfunctions, including hippocampus-dependent cognitive dysfunction (1–3). Cognitive impairments observed in hypothyroidism are associated with several neurotransmitter systems. In particular, a strong correlation has been demonstrated between the activity of thyroid hormones (THs) and the cholinergic system (4–5). Acetylcholine (ACh) is an important neurotransmitter and is crucial in hippocampal cognitive functioning. Its activity depends upon the metabolizing enzyme, acetylcholinesterase (AChE), which is responsible for the hydrolysis of acetylcholine released from presynaptic nerve terminals. Several presynaptic proteins mediate the release of neurotransmitters, including synaptotagmin-1 (syt-1) and synaptosomal-associated protein of 25 kDa (SNAP-25) (6–8). Syt-1, an abundant integral membrane protein, which is unique to small synaptic vesicles and large dense-core vesicles in the brain (9), directly interacts with SNAP-25 on the pre-synaptic membrane to facilitate neurotransmitter release. It has been demonstrated that thyroid dysfunction affects the cholinergic system and synaptic proteins in adult rats (10–13).

Thyroxine (T4) is at present the most widely accepted form of replacement therapy in hypothyroidism. However, previous studies have revealed that brain impairment induced by adult-onset hypothyroidism may not be fully restored when T4 replacement therapy returned serum THs to control levels. Clinical observations have suggested that neurocognitive functioning as well as psychological well-being may not be completely resolved in patients with hypothyroidism, despite T4 treatment (14–16). Furthermore, in morphological studies conducted by Madeira *et al*, the normalization of thyroid hormone levels in adult rats did not restore the hypothyroid-induced reduction in the number of pyramidal cells in the hippocampal CA1 region, and granule cells in the dentate gyrus (3,17). Our previous biochemical data also reported that hypothyroidism altered the expression levels of synaptic proteins in the brains of adult Sprague-Dawley (SD) rats, which concurrently, did not fully recover as a result of the standard hormone substitution therapy (13). These findings emphasize the necessity for alternative therapeutic approaches, for hypothyroidism patients that do not respond to T4 monotherapy.

Donepezil (DON) is an acetylcholinesterase inhibitor (AChEIs) that has demonstrated efficacy in improving cognitive function and providing neuroprotective effects (18), and it may have applications that extend to the treatment of hypothyroidism. Therefore, in this study, we have investigated the concentration of ACh and the activity of AChE, as well as the expression levels of syt-1 and SNAP-25 in the hippocampus of adult-onset hypothyroidism. Furthermore, the effects of T4, DON and the co-administration of both on the biochemical parameters in hypothyroid adult rats, was examined.

## Materials and methods

### Experimental animals

Eight-week-old male SD rats (~260–300g) were obtained from Nanjing Experimental Animal Center (Nanjing, China). Animals were maintained under standard laboratory conditions with a natural light-dark cycle and had free access to standard rodent chow and water. All experimental procedures adhered to the Animal Care and Use Committee of Anhui Medical University (Anhui, China) and the study was approved by the ethics committee of Anhui Medical University (Anhui, China).

Five groups of 10–12 animals were treated as follows: i) The hypothyroid group (Hypo) consisted of 12 hypothyroid rats, which were induced by including 6-n-propyl-2-thiouracil (PTU; Sigma Chemicals, Perth, WA, MO, USA) in their drinking water at a concentration of 0.05% weight/volume (w/v) for six weeks; ii) the DON group (DON) contained 11 rats, who were treated with PTU for six weeks as described above. However, from the fifth week, 0.005% (w/v) of DON (Sigma Chemicals) was added to the tap drinking water, every day for two weeks; iii) the T4 group (T4) consisted of 11 rats, who were treated with PTU for six weeks as described in the Hypo group. However, from the fifth week, they were treated with intraperitoneally injected T4 (dissolved in a saline solution, 6 μg/100 g body weight) for two weeks to restore hypothyroid animals to euthyroid status; iv) the T4 plus DON group (T4+DON) included 11 rats treated according to the same protocols as the T4 group aside from adding 0.005% (w/v) DON to the drinking water from the fifth week; v) the control group (C) contained 11 control rats, who were given the same volume of saline solution for six weeks.

### Serum hormone concentrations

Following the last delivered dose, all rats were anesthetized by chloral hydrate (350 mg/kg BW). The blood (1.5 ml) was collected from abdominal aorta and immediately centrifuged at 14,000 × g for 15 min (19). The serum was collected and quickly frozen at −20°C until the time of assay. The thyroid status of the rats was determined by measuring serum triiodothyronine (T3), T4 and thyroid stimulating hormone (TSH) levels utilizing a chemiluminescence method. All sample measurements were run in duplicate.

### Tissue preparation

The rat brains were dissected on ice following blood collection. The right hemispheres were fixed in 4% paraformaldehyde at 4°C for 7 days for immunohistochemical analysis. The hippocampus from the left hemisphere was quickly isolated and stored at −80°C for the determination of ACh content and AChE activity.

### Determination of ACh content

ACh content in hippocampus homogenates was measured using the modified method of Hestrin (20), to compare the extent of neurotransmitter levels in the hippocampus between the groups (n=11–13/group), as previously described. Briefly, 0.2 ml supernatant was mixed with 0.35 ml distilled water and was followed by adding 0.05 ml 1.5 mmol/l calabarine sulfate and 0.2 ml 1.84 mol/l trichloroacetic acid. The mixture was centrifuged at 5,000 rpm for 5 min. The ultimate supernatant (0.1 ml) was added to 0.1 ml alkaline hydroxylamine hydrochloride (equal volumes of 2.0 mol/l hydroxyl-amine hydrochloride and 3.5 mol/l sodium hydroxide) and incubated at room temperature for 15 min. This was then was reacted with 0.05 ml 4.0 mol/l HCl and 0.05 ml 0.37 mol/l ferric chloride (0.37, containing 0.1 mol/l HCl). Finally, 0.2 ml of the medium and volumes of tissue homogenates were spotted in duplicate onto 96-well microplates. Physostigmine (1.5 mmol/l) was added to the reaction mixture to inhibit the activity of AChE. Following another 2 min of incubation, the intensity of brown ferric complex was read at 540 nm on a BioTek plate reader (BioTek Instruments Inc., Winooski, VT, USA). Ach levels were expressed as μg/mg of hippocampus protein (μg/mg Prot).

### Determination of AChE activity

AChE activity in the hippocampus homogenates was determined by following the hydrolysis of acetylcholine using commercially available kits purchased from Sigma Chemicals (Poole, UK).

Briefly, the hippocampus was removed and 10% (w/v) homogenate was prepared in a sodium phosphate buffer (30 mM, pH 7.0), centrifuged at 10,000 × g for 5 min at 4°C and the supernatant was used for estimation of AChE activity. Sodium chloride (0.1 ml) was added to 0.1 ml of homogenate in a test tube and the solution was vortexed. Reference tubes were then placed in a waterbath at 60°C for 10 min in order to inactivate the cholinesterase, prior to being cooled back to room temperature. All tubes then received 1.5 ml water, 1.0 ml nitrophenol solution and 0.1 ml ACh chloride solution. Exactly 30 min from the addition of the acetylcholine chloride, 0.2 ml of each sample was transferred to cuvettes for reading in a spectrophotometer at 540 nm.

### Protein assay

Protein in hippocampal homogenates was detected using a BCA protein assay kit (Thermo Fisher Scientific, Waltham, MA, USA) according to the manufacturer’s instructions.

### Immunohistochemistry

The fixed right hemispheres were embedded in paraffin and sectioned coronally with a microtome into 6-μm-thick sections. From each rat, five sections (1/20 serial sections) of the hippocampus were selected to be mounted on polylysine-coated slides. Following deparaffinization, antigen retrieval was performed by heating the sections in 10 mM citrate buffer (pH 6.0) at 100°C for 10 min. Potential non-specific binding sites were blocked with 5% normal goat serum in phosphate-buffered saline (PBS). The sections were then incubated with the primary polyclonal antibody, rabbit anti-syt-1 (1:5,000; Chemicon, Temecula, CA, USA) or SNAP-25 (1:2,000; Sigma Chemicals, USA) at 37°C for 1 h and overnight at 4°C, followed by washes in PBS, incubation with the biotinylated secondary antibody (goat anti-rabbit IgG kit [Maixin-Bio Ltd., Fuzhou, China]) for 15 min at 37°C and further washes in PBS. Sections were further incubated with the HRP for 10 min at 37°C, washed in PBS and colored with diaminobenzidine (DAB; Maixin-Bio Ltd.) at room temperature for 5 min. Finally, sections were counterstained with hematoxylin for 3 min, dehydrated, rinsed and coverslipped with glycerin. The sections were subsequently dehydrated, rinsed and coverslipped with glycerin. With the exception of the normal goat serum-inhibition step, the sections were rinsed in PBS (three times, 5 min each) following each treatment. The negative control was processed with the same steps, however the primary antibody was replaced with PBS.

An image analysis system was used for quantitative analysis of the expression levels of yt-1 and SNAP-25. The system included MetaMorph image acquisition and processing software (JADA 801D; JEDA Science Technology Development, Co., Ltd., Nanjing, China) and a Nikon 80i microscope (Nikon, Tokyo, Japan) equipped to a computer. The layers were analyzed from different subfields of the hippocampus, including the stratum oriens (SO), stratum radiatum (SR) and stratum lacunosum-moleculare (SLM) in the CA1; the SO, stratum lucidum (SL) and SR in the CA3, polymorphic layer (PL) and molecular layer (ML) in the dentate gyrus (DG). An image of the entire hippocampal structure was obtained initially at a low magnifcation (x40) and subsequent images at a higher magnification (x200), in various subfields of the hippocampus, were acquired according to the size of each subfeld: three images in CA1 for SO and SR; one image in CA3 and DG-PL and two images in DG-ML and CA1-SLM. The digital data were then exported into the MetaMorph software for analysis and processing. The average optical density (OD) represented the intensity of the immunohistochemical staining.

### Statistical analysis

All statistical analysis was performed using SPSS 13.0 software (SPSS, Inc., Chicago, IL, USA). Values are expressed as the mean ± standard error (SEM). The data were analyzed by one-way analysis of variance (ANOVA) using least-significant difference for post hoc analysis. P<0.05 was considered to indicate a statistically significant result.

## Results

### Serum concentrations of the hormones

Serum T3, T4 and TSH concentrations are presented in Table I. The serum T3 and T4 levels were significantly lower (P<0.001) and TSH levels were higher (P<0.001) in SD rats of the Hypo and DON groups, compared with the controls. T4 and T4+DON treatment restored T3, T4 and TSH levels, which were not significantly different from the control values (P>0.05).

### Content of ACh in the rat hippocampus

**Figure 1:**
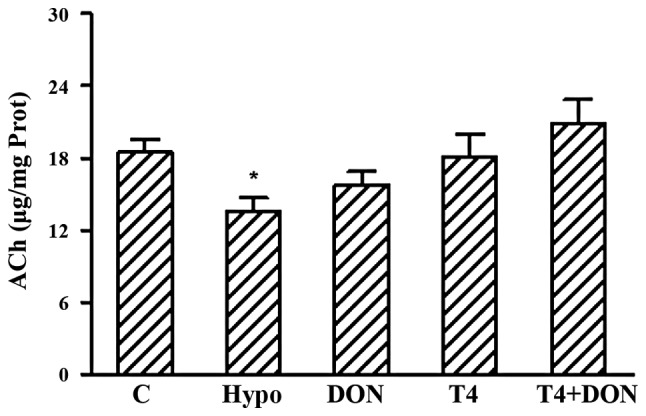
Concentration of hippocampal ACh from Hypo, T4, DON, T4+DON and C groups (n=10–12). Homogenates were extracted from the hippocampus of each rat. Hypothyroidism induced a significant decrease in ACh content in the hippocampus, and the DON (0.005%), T4 (6 μg/100 g body weight) or combined treatment (T4+DON) restored the Ach levels to the control value. Data shown are the mean ± SEM of three independent experiments. C, control group; Hypo, hypothyroid group; DON, hypothyroid rats treated with 0.005% (w/v) DON; T4, hypothyroid rats treated with 6 μg T4/100 g BW; T4+DON, hypothyroid rats treated with 6 μg T4/100 g BW beside adding 0.005% (w/v) DON to the drinking water. ^*^P<0.05, vs C. ACh, acetylcholine; T4, thyroxine; DON, donepezil; Prot, hippocampus protein; SEM, standard error of the mean; w/v, weight/volume.

Alkaline hydroxylamine colorimetry was performed to detect the content of Ach in the hippocampus of the rats among the groups. ACh content in the hippocampus is illustrated in Fig. 1. Our results demonstrated that the amount of ACh was significantly decreased by 27% in the hypothyroid rats (P=0.027) and the content was restored to control values (P=0.212, 0.860 and 0.255, respectively) by DON, T4 or T4+DON treatment.

### AChE activity in rat hippocampus

**Figure 2:**
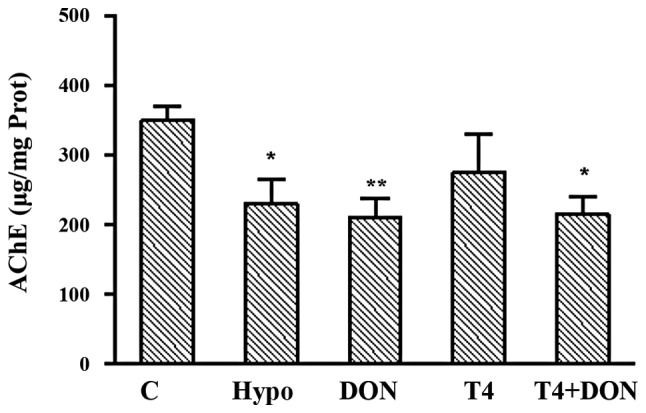
The AChE activity in the hippocampus of rats from Hypo, T4, DON, T4+DON and C groups (n=10–12), homogenates were extracted from the hippocampus of each rat. Data shown are the mean ± SEM of three independent experiments. C, control group; Hypo, hypothyroid group; DON, hypothyroid rats treated with 0.005% (w/v) DON; T4, hypothyroid rats treated with 6 μg T4/100 g body weight; T4+DON, hypothyroid rats treated with 6 μg T4/100 g body weight beside adding 0.005% (w/v) DON to the drinking water; C, Control group. ^*^P<0.05; ^**^P<0.01 compared with C. T4, thyroxine; DON, donepezil; AChE, acetylcholinesterase; SEM, standard error of the mean; w/v, weight/volume.

AChE activity in the hippocampus is illustrated in Fig. 2. The results demonstrated that AChE activity was significantly decreased by 34, 40 and 39% (P=0.028, 0.014 and 0.016, respectively) in the Hypo, DON and T4+DON groups, as compared with the rats in the C group, and T4 administration restored the AChE activity to the control value.

### Protein levels of syt-1 and SNAP-25 in the hippocampus. Immunohistochemistry

**Figure 3:**
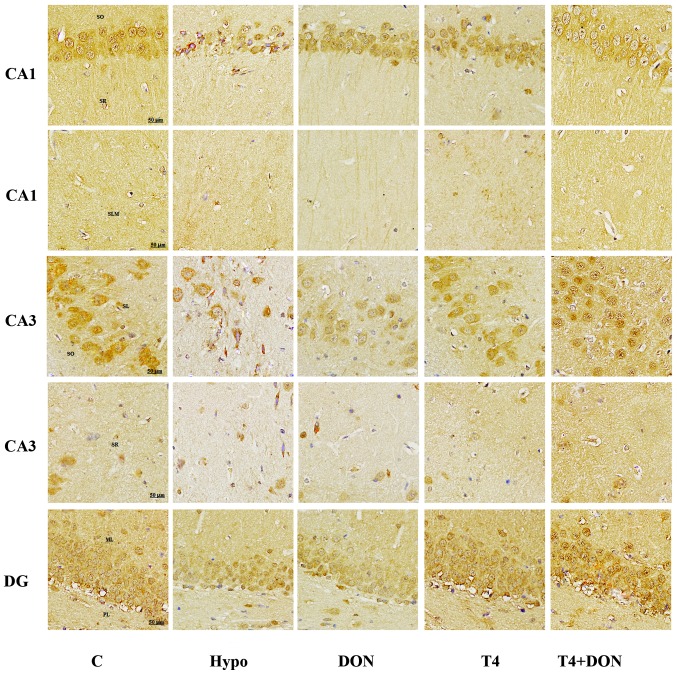
Photomicrographs of coronal sections showing syt-1 immunoreactivity in CA1, CA3 and DG subregions of hippocampus of rats from Hypo, T4, DON, T4+DON and C groups (n=10–12). Distinct punctate spots of reaction product were observed in every layer of CA1, CA3 and DG subregions; note that slight decrease in overall staining intensity of CA1-SO, CA1-SR, CA3-SL, CA3-SR and DG-ML in the Hypo, DON and T4 groups was observed; the overall staining intensity was equal in CA1-SLM, CA3-SO and DG-PL of five groups; magnification: ×400, scale bar = 50 μm. Hypo, hypothyroid group; DON, hypothyroid rats treated with 0.005% (w/v) DON; T4, hypothyroid rats treated with 6 μg T4/100 g body weight; T4+DON, hypothyroid rats treated with 6 μg T4/100 g body weight beside adding 0.005% (w/v) donepezil to the drinking water; C, Control group. Syt-1, synaptotagmin-1; T4, thyroxine; DON, donepezil; DG, dentate gyrus; SO, stratum oriens; SR, stratum radiatum; SLM, stratum lacunosum-moleculare; SL, stratum lucidum; ML, molecular layer; PL, polymorphic layer; w/v, weight/volume.

**Figure 4:**
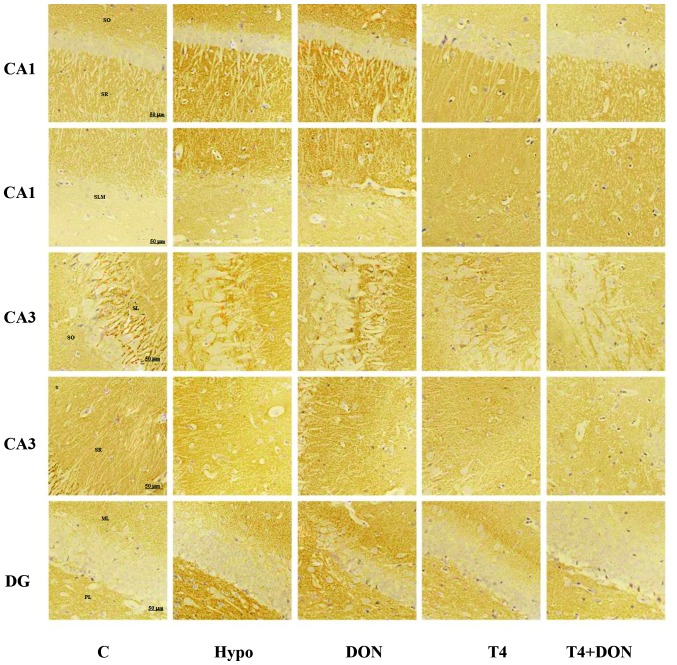
Photomicrographs of coronal sections showing SNAP-25 immunoreactivity in CA1, CA3 and DG subregions of hippocampus of rats from Hypo, T4, DON, T4+DON and C groups (n=10–12). Distinct punctate spots of reaction product were observed in every layer of CA1, CA3 and DG subregions; note that the staining for SNAP-25 was more intense in CA3-SO, CA3-SR and in all layers of CA1 and DG of Hypo and DON groups, and the overall staining intensity was equal in CA3-SL of five groups; magnification, ×400; scale bar: 50 μm. C, control group, Hypo, hypothyroid group; DON, hypothyroid rats treated with 0.005% (w/v) donepezil; T4, hypothyroid rats treated with 6 μg T4/100 g body weight; T4+DON, hypothyroid rats treated with 6 μg T4/100 g body weight beside adding 0.005% (w/v) donepezil to the drinking water. Syt-1, synaptotagmin-1; T4, thyroxine; SNAP-25, synaptosomal-associated protein of 25 kDa; DON, donepezil; DG, dentate gyrus; SO, stratum oriens; SR, stratum radiatum; SLM, stratum lacunosum-moleculare; SL, stratum lucidum; ML, molecular layer; PL, polymorphic layer; w/v, weight/volume.

Representative photomicrographs of the immuno-labeled syt-1 and SNAP-25 in subfields (CA1, CA3 and DG) of the hippocampus from different groups are demonstrated in Fig. 3 and Fig. 4, respectively. The immunoreactivity distributions of the proteins were similar among these groups. Punctate spots of reaction product were distributed in every layer of the CA1, CA3 and DG subregions of the hippocampus.

The OD value corresponding to syt-1 immunoreactivity in each stratum of hippocampal subfields is summarized in Table II. The OD values of five layers of CA1, CA3 and DG subfields in the Hypo, DON and T4 groups were significantly lower compared with the corresponding layers in the C group (P<0.05), including CA1-SO, CA1-SR, CA3-SL, CA3-SR and DG-ML. In the T4+DON group, the OD values in all layers were similar to those in the C group.

The OD value of SNAP-25 immunoreactivity in each stratum of hippocampal subfields is also analyzed and summarized in Table III. It was identified that the OD values of SNAP-25 in CA3-SO, CA3-SR and in all layers of CA1 and DG, were significantly higher in Hypo and DON groups compared with the corresponding layers in the C group (P<0.01). In the T4 group, the OD values were significantly higher compared with the C group in all layers of CA1 and CA3-SO (P<0.01). In the T4+DON group, the OD values in all layers were similar to that observed in the C group.

## Discussion

In the present study, it has been demonstrated that there are significant decrements of Ach levels, as well as AChE activity, in the hippocampus of adult-onset hypothyroidism. A decrease in AChE activity has previously been identified in various brain regions, including the cerebellum, frontal cortex, subcortex and medulla of hypothyroid adult rats (11,21). The effects induced by hypothyroidism on rat hippocampal Ach and AChE remain largely unknown. The hippocampus has been reported to be rich in cholinergic synapses (22) and thus hypothyroidism may result in hippocampal cholinergic neuronal impairment. This may be because the hippocampus is vulnerable to the deleterious effects of hypothyroidism at developmental and adult stages (23), which subsequently leads to an insufficient synthesis of ACh and its enzyme protein. Indeed, this hypothesis is consistent with studies that have reported decreased choline acetyltransferase (ChAT; involved in the synthesis of ACh) activity and protein concentration in hypothyroidism (11,24–25). Alternatively, the decrease in AChE activity may also result from changes in the turnover of the enzyme proteins in hypothyroidism (26–27).

Using immunohistochemical analysis, the results indicated that the effects of adult-onset hypothyroidism on syt-1 and SNAP-25 are different, although these proteins are required for neurotransmitter exocytosis. The relative expression of syt-1 was decreased in certain layers of hippocampus of hypothyroid rats compared with the controls, and the simultaneous upregulation of SNAP-25 expression was also observed. The results obtained are consistent with our previous study (13). The mechanism underlying this differential regulation remains elusive. However, it has been hypothesized that the reduced expression of syt-1 may be due to the reduced expression of TH receptors as well as a decrease in the number and/or volume of neurons in the hippocampus associated with hypothyroidism (3,17,28), considering it is an abundant constituent of synaptic vesicles (9). While SNAP-25 overexpression may be a compensatory mechanism against adult-onset hypothyroidism, functioning to rescue vesicle exocytosis.

The changes in hippocampal ACh content and AChE activity observed in the present study, during adulthood, appear to be reversible, as decreased ACh levels and AChE activity were restored to control values following T4 treatment. Similar results were observed in rats studied during the critical stages of development, in which treatment of 26-day-old neonatally thyroidectomy rats with thyroid hormone was revealed to virtually replenish the levels of the brain ACh to control values (29). Therefore, it may be that ACh and its enzyme protein are under direct thyroid hormone control. Evidence from tissue culture experiments indicate that as T3 media concentrations approach the level of total TH in the blood, the cholinergic enzymatic activities of ChAT and AChE were markedly enhanced (26). Of note, DON treatment (via drinking water) for 2 weeks also increased the levels of ACh in the hippocampus of adult hypothyroid rats to meet the values recorded from the control rats. This result is consistent with previous studies demonstrating that AChEIs, including physostigmine and galantamine, increase ACh levels (30–31). Therefore it is probable that DON increases ACh levels through a well-established mechanism of preventing its enzymatic degradation and thus prolonging the availability of ACh once it is released into the synapse.

By the administration of T4, it was observed that hypothyroidism-induced downregulation of the syt-1 protein and upregulation of the SNAP-25 protein was restored in the hippocampus. However, the parameters did not reach the control level. Previous animal studies have also reported that normal ranges of hormone substitution did not reverse the reduction in protein kinase C-γ and syt-1 levels in hypothyroid rats (13,32). However, T4 and DON treatment in combination, did result in the normalization of the hippocampal syt-1 and SNAP-25 levels in the adult hypothyroid rats. This suggests that DON treatment resulted in amelioration of hypothyroidism-induced synaptic protein impairment, although the exact mechanism underlying this regulation remains elusive. In recent years, the AChEIs have demonstrated neuroprotective effects. DON has been reported to ameliorate synaptic loss and tau pathology in the tauopathy mouse model, which may be due to ACh-induced anti-inflammatory effects (18). In addition, it has been suggested that DON slows the progression of hippocampal atrophy in patients with Alzheimer’s disease (33–34) and reduce cell death induced by exogenous cytotoxins, including glutamate, amyloid-β, okadaic acid and carbon monoxide gas (35–39). The observed normalization of the synaptic proteins in hypothyroidism may also occur as a result of DON-induced neuroprotection against hippocampal impairment, leading to an alteration in the synthesis of these synaptic proteins. To elucidate the mechanisms underlying the neuroprotective effects of DON on hypothyroidism, further investigation is required.

In conclusion, this study demonstrated that hypothyroidism induces alterations of hippocampal Ach level and AChE activity, as well as syt-1 and SNAP-25 expression, in adult rats. Ach levels and AChE activity were restored by T4 administration, while expression of syt-1 and SNAP-25 did not reach the control level. Significantly, co-administration of T4 and DON led to normalization of the syt-1 and SNAP-25 levels, suggesting that DON treatment may facilitate the recovery of synaptic protein impairment induced by hypothyroidism. Nevertheless, the efficacy of DON treatment and the molecular mechanisms underlying this regulation require further exploration to be of therapeutic value.

## Figures and Tables

**Table I:** Serum T3, T4 and TSH levels in five groups.

Group	Number	T3 (nmol/l)	T4 (nmol/l)	TSH (μIU/ml)
C	12	0.83±0.03	49.81±1.08	1.02±0.14
Hypo	11	0.60±0.03*	18.19±1.72*	19.78±3.01*
DON	11	0.57±0.02*	18.58±0.91*	19.55±3.29*
T4	10	0.83±0.08	52.42±1.92	1.21±0.32
T4+DON	11	0.77±0.07	52.71±2.04	1.07±0.15

Data are expressed as the mean ± SEM. C, control group; Hypo, hypothyroid group; DON, hypothyroid rats treated with 0.005% (w/v) DON; T4, hypothyroid rats treated with 6 μg T4/100 g body weight; T4+DON, hypothyroid rats treated with 6 μg T4/100 g BW beside adding 0.005% (w/v) DON to the drinking water.

* P<0.01, vs. C.

T3, triiodothyronine; T4, thyroxine; DON, donepezil; THS, thyroid stimulating hormone; SEM, standard error of the mean; w/v, weight/volume.

**Table II:** syt-1 in different layers of each subfield in the hippocampus.

Subfield	Stratum	C	Hypo	DON	T4	T4+DON
CA1	SO	5.06±0.15	3.49±0.13**	3.85±0.14**	4.09±0.20**	4.79±0.16
	SR	4.75±0.19	3.19±0.14**	3.59±0.18**	3.70±0.29*	4.29±0.28
	SLM	4.31±0.09	3.91±0.16	3.99±0.11	3.99±0.05	4.42±0.09
CA3	SO	5.01±0.18	4.55±0.17	4.71±0.08	4.87±0.10	4.98±0.14
	SL	4.85±0.10	3.94±0.04**	3.95±0.05**	4.23±0.07**	4.71±0.08
	SR	4.94±0.05	3.90±0.07**	4.05±0.05**	4.46±0.09**	4.81±0.06
DG	ML	4.75±0.06	4.09±0.03**	4.18±0.09**	4.37±0.05**	4.61±0.05
	PL	3.76±0.05	3.51±0.03	3.57±0.10	3.67±0.07	3.72±0.06

Data (mean ± SEM) are expressed as the average OD of syt-1 immunoreactivity (n=10–12). C, control group; Hypo, hypothyroid group; DON, hypothyroid rats treated with 0.005% (w/v) DON; T4, hypothyroid rats treated with 6 μg T4/100 g body weight; T4+DON, hypothyroid rats treated with 6 μg T4/100 g BW beside adding 0.005% (w/v) donepezil to the drinking water.

* P<0.05,

** P<0.01, vs. C.

Syt-1, synaptotagmin-1; T4, thyroxine; DON, donepezil; DG, dentate gyrus; SO, stratum oriens; SR, stratum radiatum; SLM, stratum lacunosum-moleculare; SL, stratum lucidum; ML, molecular layer; PL, polymorphic layer; SEM, standard error of the mean; OD, optical density; w/v, weight/volume.

**Table III:** SNAP-25 in different layers of each subfield in the hippocampus.

Subfield	Stratum	C	Hypo	DON	T4	T4+DON
CA1	SO	3.55±0.11	4.82±0.08**	4.49±1.13**	4.39±0.05**	3.80±0.12
	SR	3.72±0.15	4.64±0.14**	4.43±0.18**	4.34±0.12*	4.16±0.13
	SLM	3.52±0.11	4.36±0.12**	4.26±0.09**	4.00±0.12*	3.77±0.11
CA3	SO	4.07±0.06	5.04±0.10**	5.02±0.10**	4.83±0.06**	4.40±0.11
	SL	3.63±0.12	4.10±0.12	4.09±0.15	3.92±0.11	4.07±0.09
	SR	3.67±0.14	4.36±0.09**	4.29±0.12**	3.95±0.09	3.80±0.09
DG	ML	3.61±0.10	4.69±0.13**	4.57±0.06**	4.00±0.09	3.81±0.09
	PL	3.66±0.11	4.75±0.04**	4.46±0.09**	3.95±0.06	3.85±0.05

Data (the mean ± SEM) are expressed as the average OD of SNAP-25 immunoreactivity (n=10–12). C, control group; Hypo, hypothyroid group; DON, hypothyroid rats treated with 0.005% (w/v) DON; T4, hypothyroid rats treated with 6 μg T4/100 g BW; T4+DON, hypothyroid rats treated with 6 μg T4/100 g body weight beside adding 0.005% (w/v) donepezil to the drinking water.

* P<0.05,

** P<0.01, vs. C.

SNAP-25, synaptosomal-associated protein of 25 kDa; DG, dentate gyrus; SO, stratum oriens; SR, stratum radiatum; SLM, stratum lacunosum-moleculare; SL, stratum lucidum; ML, molecular layer; PL, polymorphic layer; OD, optical density; w/v, weight/volume.
